# Stemming the Flow: Causes and Solutions for Blood and Blood Component Wastage in a Tertiary Care Hospital

**DOI:** 10.7759/cureus.59493

**Published:** 2024-05-01

**Authors:** Hari Haran, Suresh Kumar I, Sahayaraj J

**Affiliations:** 1 Transfusion Medicine, Saveetha Medical College and Hospital, Saveetha Institute of Medical and Technical Sciences, Chennai, IND

**Keywords:** inventory management, quality indicator, transfusion transmitted infection, expiry, wastage

## Abstract

Aim

This study aims to analyze the discard rates and causes of blood and blood component wastage in a hospital transfusion service and identify strategies for improvement.

Methodology

We conducted a retrospective study reviewing data from the Department of Transfusion Medicine over five years. We calculated discard rates for different blood components and categorized the reasons for discard.

Results

The overall discard rate was 18%. Platelets were the most commonly discarded component (91.6%), followed by plasma (4.4%) and packed red blood cells (3.8%). Expired shelf life was the most frequent reason for discard (97%), followed by transfusion-transmitted infection (TTI) reactivity (2.9%), and bag breakage (0.01%).

Conclusions

Platelets were the most commonly discarded component, and expiry due to non-utilization was the main cause. Implementing strategies such as improved blood utilization guidelines, staff training, and inventory management can help reduce wastage.

## Introduction

The transfusion of blood and blood components has become a part of patient management in modern medical practices [1]. Blood is the precious and irreplaceable liquid organ that serves as support for many patients requiring transfusions due to trauma, surgery, and various other medical conditions. Blood is irreplaceable and should be utilized wisely with minimum wasting [2].

Usage of blood components is more than whole blood usage in all medical and surgical cases in this era. One whole blood unit donated can be separated into three blood components, thereby saving three patients [3].

As a scarce resource, ensuring its wise and optimal utilization remains dominant. Unfortunately, a sizable portion of donated blood is discarded, hindering its reach to ones in need and posing ethical and financial concerns. Therefore, blood transfusion services (BTS) play a vital part and are accountable for providing high-quality, safe, and secure blood products [1].

The discard rate of blood components reflects the overall efficiency, performance, and planning of a BTS [1,2]. Analyzing those rates and understanding the underlying causes are vital for enforcing targeted guidelines to minimize wastage and optimize blood utilization practices [3,4]. This retrospective study aimed to investigate the discard rates and contributing factors within our hospital over five years (2019-2023).

By analyzing the data, we aimed to recognize the gray areas for improvement and suggest evidence-based strategies to minimize blood wastage. This, in turn, may contribute to improving the efficiency, performance, and effectiveness of our transfusion service, ultimately ensuring better patient care and optimizing the utilization of this lifesaving resource.

## Materials and methods

A retrospective study was conducted in a tertiary care hospital in south India, over five years, from January 2019 to December 2023. Data relating to the collection, usage, and discard of blood and its components were obtained from the records of the Department of Transfusion Medicine.

Inclusion criteria

The present study includes blood units discarded for different reasons, including transfusion-transmitted infection (TTI) reactivity, expired component/ expiry, less quantity (LQ), leakage/breakage, and clotted bag. Blood components such as packed red blood cells (PRBCs), platelet concentrate, fresh frozen plasma (FFP), and cryoprecipitate (Cryo), are prepared regularly from 350 mL blood bags under all aseptic conditions, as advised by the National Accreditation Board for Hospitals and Healthcare Providers (NABH), Third Edition, and the National AIDS Control Society (NACO).

Statistical tool

Data were compiled and analyzed using Microsoft Excel.

The reasons for discarding blood units were broadly classified into donation, processing, storage, and post-issue related, to determine the type of intervention required to minimize the waste. The causes for the discarded units [3,4] during this period were tabulated and analyzed (Table 1). 

**Table 1:** Causes for discarding blood components.

Donation related	Processing related		Storage related	Post issue
Under/Overcollection	Bag breakage/leakage during centrifugation		Bag breakage during storage/thawing	Unused after issue
Confidential unit exclusion (CUE)	Cell contamination		Storage temperature not appropriate	Bag leak
Lipemia	Low volume		Expiry of shelf life	Precipitates
Antibody positive	Hemolysis during leucofiltration		Hemolysis	Cold chain not maintained
Presence of clot			Absent swirling	
Transfusion-transmitted infection (TTI) reactive status				

The following formula was used to calculate the discard rate:

Discard rate = Number of blood and blood components discarded/Total number of blood and blood components issued × 100.

Ethical clearance

The present study was retrospective. Donor details were kept confidential. Local management’s clearance was taken before data compilation. Only data related to blood donation and blood discard were retrieved from the registers and analyzed, and institutional ethical committee clearance was obtained for this study.

## Results

During the five-year study period spanning from 2019 to 2023, a comprehensive analysis was conducted on collecting and utilizing blood components. The total count of whole blood units collected during this period amounted to 17,813. Among these, a fraction of 192 units, which accounts for approximately 1.07%, were deemed incomplete collections (INC) due to occurrences of donor reactions and low flow rates.

After the identification of INC units, blood processing ensued, leading to the preparation of blood components such as PRBCs, FFP, platelet concentrate, and cryoprecipitate from the remaining 17,621 viable blood units. It is imperative to note that adhering strictly to a policy of 100% component therapy, no whole blood units were utilized.

**Figure 1:**
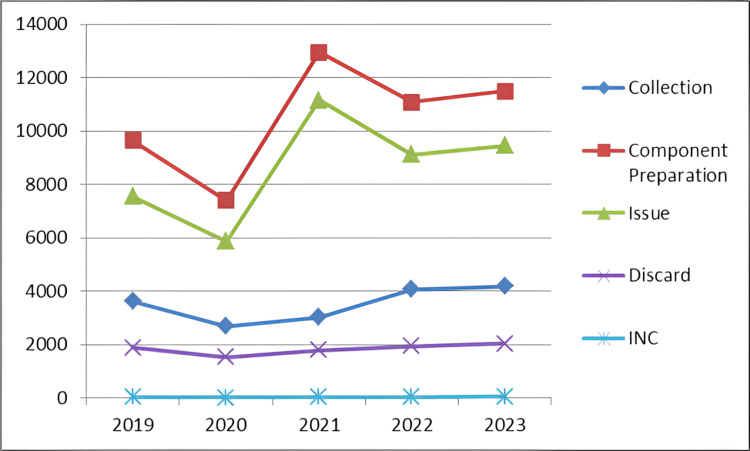
Trend of blood component collection, preparation, issue, and discard. INC, incomplete collection

The totality of components derived from the 17,621 units amounted to an impressive 52,553 units. Of these prepared components, 43,152 units, constituting approximately 82% of the total, were effectively issued for various medical procedures. Regrettably, a notable portion of 9,201 units, representing around 18% of the total, were inevitably discarded (Figure 1 and Table 2).

**Table 2:** Number of blood units collected, components prepared, issued, and discarded over the past five years.

Year	Collection (no. of units)	Component preparation (no. of units)	Issue (no.of units)	Discard (no. of units)
2019	3,631	9,636	7,554	1,882
2020	2,696	7,403	5,867	1,536
2021	3,023	12,955	11,152	1,803
2022	4,075	11,073	9,123	1,950
2023	4,196	11,486	9,456	2,030
Total	17,621	52,553	43,152	9,201

**Figure 2:**
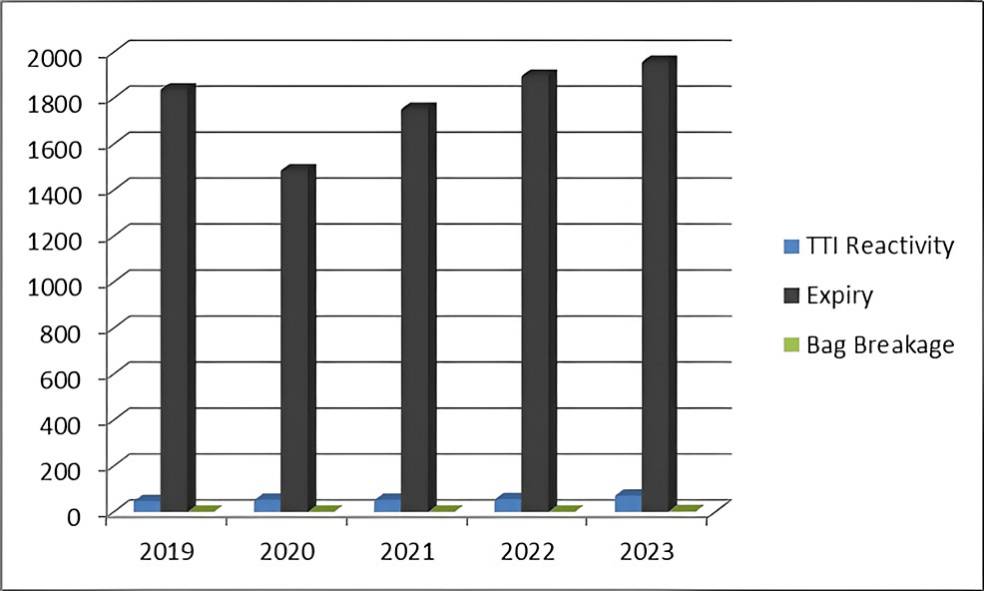
Reasons for discarding blood components. TTI, transfusion-transmitted infections

Upon closer examination of the reasons behind this discard, it became apparent that the primary cause, accounting for a staggering 97% of the instances, was expiry. Trailing behind were TTIs and bag breakage, comprising 2.95% and 0.03% of the cases, respectively (Figure 2 and Table 3).

**Table 3:** Number of blood components discarded. TTI, transfusion-transmitted infection; PRBC, packed red blood cells; Cryo, cryoprecipitate

Year	Reason for discard (No. of units)			Type of blood components (No. of units)				Total discard (No. of units)
	TTI reactivity	Expiry	Bag breakage	PRBC	Platelet	Plasma	Cryo	
2019	46	1,836	0	52	1,774	52	4	1,882
2020	51	1,485	0	73	1,373	88	2	1,536
2021	51	1,752	0	80	1,611	104	8	1,803
2022	54	1,896	0	62	1,811	75	2	1,950
2023	70	1,957	3	78	1,869	81	2	2,030
Total	272	8,926	3	345	8,438	400	18	9,201

**Figure 3:**
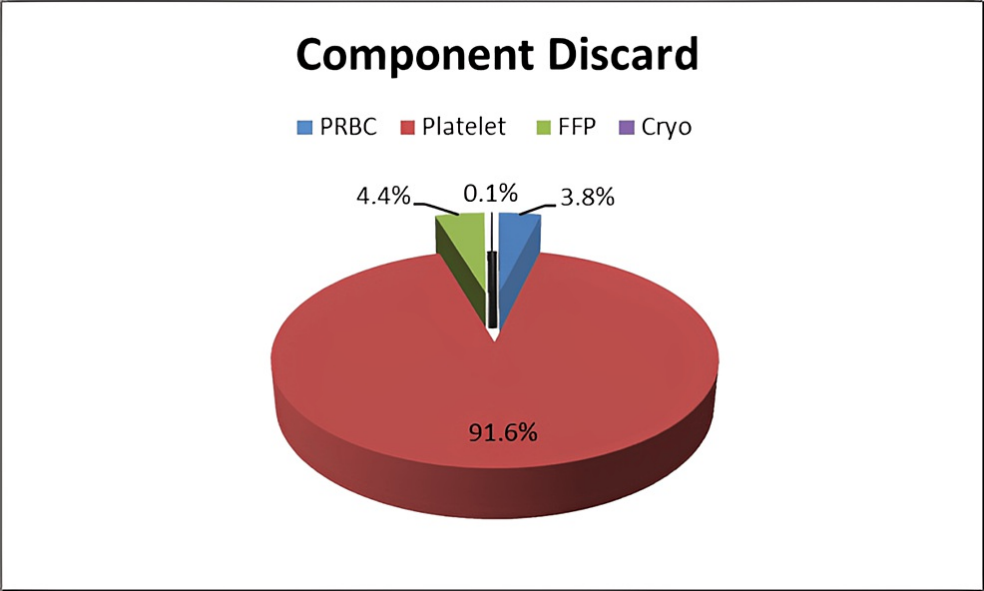
Blood component discard rate in the past five years. PRBC, packed red blood cells; FFP, fresh frozen plasma; Cryo, cryoprecipitate

**Figure 4:**
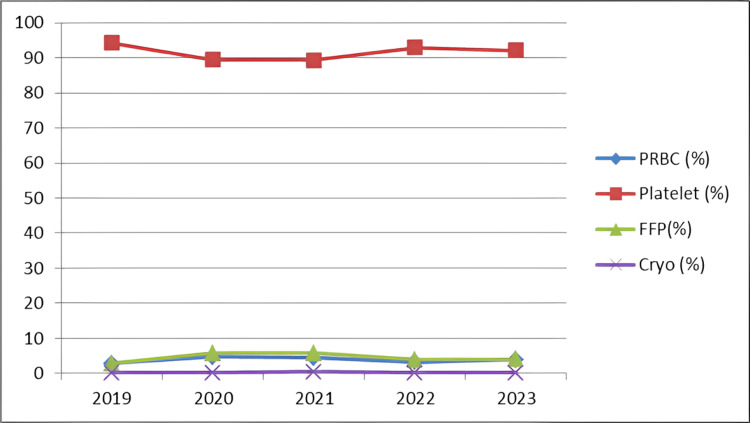
Trend of blood components discarded over the past five years. PRBC, packed red blood cells; FFP, fresh frozen plasma; Cryo, cryoprecipitate

Further delving into the composition of the discarded units, it was observed that a diverse array of blood components contributed to the discarded total. Specifically, 3.8% of the discarded units were PRBCs, 4.4% were plasma, 0.19% constituted cryoprecipitate, and the overwhelming majority, amounting to 91.6%, were platelets (Figures 3-4 and Table 4).

**Table 4:** Component-wise discard rate (%). PRBC, packed red blood cell; FFP, fresh frozen plasma; Cryo, cryoprecipitate

Year	PRBC (%)	Platelet (%)	FFP (%)	Cryo (%)
2019	2.76	94.26	2.76	0.21
2020	4.75	89.38	5.72	0.13
2021	4.43	89.35	5.76	0.44
2022	3.17	92.87	3.84	0.1
2023	3.84	92.06	3.99	0.09
Total	3.79	91.584	4.414	0.194

## Discussion

In this study, the total discard rate was 18%, while it was less than 20% in a study conducted by Patil et al. [5]. The reasons for discarding were primarily the expiry of shelf life, which is the most common cause, constituting 97% of the total discards (Table 5). Following expiry, TTI reactivity was the second most common cause, constituting around 2.9% of the total discards. This percentage was significantly lower compared to findings by Kumar et al. [4] (33.8%) and Patil et al. [5] (33%). 

**Table 5:** Comparison of discard rates in this study with those of other studies and reasons for discarding blood units.

Literature	Total discard (%)	Expiry (%)	TTI (%)	Bag breakage (%)	
Kanani et al. [2]	7.0	43.4	11.3	13.7	
Kumar et al. [4]	8.4	57.8	33.8	3.0	
Patil et al. [5]	20.6	53	33.0	3.4	
Anitha et al. [6]	4.6	21.9	63.6	10.8	
Luhar et al. [7]	2.96	21.06	35.06	13.84	
Kulkarni et al. [8]	68.33	14.5	54.75	27.0	
Suresh et al. [9]	7.0	7.5	37.9	1.6	
Bobde et al. [10]	8.2	51.4	19.5	34.5	
Deb et al. [11]	14.6	82.8	17.2	-	
Sharma et al. [12]	8.69	54.5	20	25.6	
This study (2023, South India)	18	97	2.9	0.01	

TTI reactivity, while a lower contributor (2.9%), highlights the importance of stringent donor screening and adherence to pre-donation protocols. 

Damage during processing/bag breakage (0.1%) is much less compared to 10.8% and 13.84% in studies conducted by Anita et al. [6] and Luhar et al., respectively [7]. However, it is very minimal, indicating a potential area for improvement in handling procedures.

Of the discarded blood components, 91.6% were platelets, 3.8% were PRBC and 4.4% were plasma. The average discard rate of whole blood in the present study was 1.07%, which was much less compared to Kulkarni et al. [8] (23.23%), and lesser than the studies conducted by Suresh et al. [9] (5.7%) and Bobde et al. [10] (6.63%).

Conversely, our study identifies a higher expiration rate which is similar to Kumar et al. [4] (57.8%), Deb et al. [11] (82.8%), and Sharma et al. [12] (54.5%). This is attributed to the decreased utilization of platelet concentrate, as a consequence of the prevailing preference for single-donor platelets over random donor platelets, coupled with the widespread adoption of 100% component therapy, resulting in a surplus of platelet concentrates within the inventory.

Study limitation

This design utilizes current medical records, limiting the exploration of modifiable factors affecting reasons for discarding. Conducting prospective data collection could enhance accuracy by investigating the influential factors behind the causes.

## Conclusions

Our study revealed that platelet concentrates were the most common waste products. This is mainly because 97% of discard were due to the expiry of shelf life. Thus, improved inventory management and forecasting practices are needed. Platelet management can be optimized using targeted approaches such as point-of-care and individual ordering of platelets.

In addition, establishing a well-organized blood donation program can reduce the wastage of blood units due to expiration. Collaboration between donors, administrative staff, and end users is essential to ensure transfusion efficiency.

Since blood is a valuable resource, minimizing wastage through regular internal audits, close cooperation with hospital departments and transfusion service personnel, appropriate management of inventory management system, strict selection criteria, and comprehensive history-taking practices are needed.
